# Fat Mass Index Associated with Blood Pressure Abnormalities in Children with Chronic Kidney Disease

**DOI:** 10.3390/children8080621

**Published:** 2021-07-22

**Authors:** Chien-Ning Hsu, Pei-Chen Lu, You-Lin Tain

**Affiliations:** 1 Department of Pharmacy, Kaohsiung Chang Gung Memorial Hospital, Kaohsiung 833, Taiwan; cnhsu@cgmh.org.tw; 2 School of Pharmacy, Kaohsiung Medical University, Kaohsiung 807, Taiwan; 3 Department of Pediatrics, Kaohsiung Chang Gung Memorial Hospital, Chang Gung University College of Medicine, Kaohsiung 833, Taiwan; latina@cgmh.org.tw

**Keywords:** cardiovascular disease, ambulatory blood pressure monitoring, fat mass index, children, dual-energy X-ray absorptiometry, chronic kidney disease, hypertension

## Abstract

Cardiovascular disease (CVD) risk factors are present early in life in children with chronic kidney disease (CKD), consequently cardiovascular morbidity presents in early adulthood. However, risk factors of CVD have been rarely addressed in children with early stage of CKD. This study included 63 children and adolescents aged 8- to 18 years-old with CKD stage G1–G4. Cardiovascular assessments consisted of 24-h ambulatory blood pressure monitoring (ABPM), arterial stiffness index, and echocardiography. We also applied dual-energy x-ray absorptiometry (DXA) scanning to analyze percentage body fat (PBF), lean body mass index (LBMI), fat mass index (FMI), and the android to gynoid fat ratio (A/G ratio). Up to 63.5% of CKD children had abnormal changes in BP detected by ABPM. CKD children with abnormal ABPM were older, had higher numbers of CKD stage G2 to G4, hyperuricemia, obesity, and higher FMI z-score and A/G ratio compared to individuals with normal ABPM (all *p* < 0.05). Among these factors, only FMI z-score showed an independent association with abnormal ABPM using multivariate logistic regression analysis (*p* = 0.037). Our data highlight that body fat plays a key role for an abnormal ABPM in CKD children. The assessment of FMI may have clinical utility in discriminating CV risk in children and adolescents with early stages of CKD.

## 1. Introduction

Patients with chronic kidney disease (CKD) exhibit a marked risk for cardiovascular (CV) comorbidities and mortality [1], whereas major CV events are uncommon in children with CKD [2]. Hypertension is an early sign of CVD and is the most common complication of childhood CKD [2]. Using 24-h ambulatory blood pressure monitoring (ABPM), we and others have shown that hypertension is present in more than one-half of children with CKD, which occurs even in early stages [3,4]. However, subclinical CVD presenting in children is hardly detectable by conventional methods [5]. Left ventricle mass index (LVMI), ambulatory arterial stiffness index (AASI), and carotid intima-media thickness (cIMT) have been considered as surrogate markers for CVD in children with CKD [6,7,8].

Similar to adults, risk factors of CVD among children with CKD are also related to the traditional risks [2]. Childhood obesity has become a public issue as its prevalence increases at an alarming rate [9]. Obesity is associated with increased risk of developing CVD in people without CKD, but the effect of obesity in people with CKD is inconclusive [10,11,12]. Despite obesity in children being defined as a body mass index (BMI) at or above the 95th percentile for age and sex [13], BMI does not measure body fat directly. Currently, dual-energy x-ray absorptiometry (DXA) is increasingly used to measure body composition in terms of fat and fat-free mass [14]. Fat mass index measured by DXA has been shown to correlate with CV risk factors in CKD adults [14] and overweight/obese children [15]. Nevertheless, it remains unclear whether these measures are associated with CVD risk in pediatric patients with CKD.

The purpose of our study, therefore, was to assess body composition parameters with DXA and find out their associations with ABPM and other surrogate markers (i.e., LVMI, cIMT, AASI) in pediatric CKD.

## 2. Materials and Methods

### 2.1. Patients and Study Design

All study procedures were performed in accordance with the ethical standards laid down in the 1964 Declaration of Helsinki and its later amendments. The prospective cohort study was approved by the Institutional Review Boards (IRB) of the Chang Gung Medical Foundation at Taoyuan, Taiwan (201601181A3). A total of 101 children and adolescents aged 3 to 18 years with CKD stage G1–G4 attending the pediatric clinic at Kaohsiung Chang Gung Memorial Hospital was enrolled between December 2016 and October 2018. All of the participants parents gave their written informed consent. CKD was defined by kidney damage or reduced kidney function over at least three months [16]. Kidney damage included functional abnormalities or structural abnormalities identified by kidney biopsy, imaging studies, or urinary sediment [16]. Renal function was determined by estimated glomerular filtration rate (eGFR) using the Schwartz formula on the basis of body height and blood creatinine (Cr) level [17]. Participants were classified into five CKD stages according to their eGFR [16]. Patients were excluded, if they (1) were pregnant; (2) had history of congenital heart disease; (3) had eGFR < 15 mL/min/1.73 m^2^, on dialysis maintenance, or ever received renal transplantation; or (4) were incapable of consenting to the follow-up protocol or cooperated with the procedures. All recruited patients were followed up to progression to end-stage kidney disease.

### 2.2. Data and Specimens

The following assessments were performed on each participant at the same clinic visit: (1) history taking and physical examination; (2) anthropometry; (3) office BP and ABPM measurements; and (4) laboratory investigations. The congenital anomalies of the kidney and urinary tract (CAKUT) includes a wide range of structural anomalies like renal agenesis, posterior urethral valves, horseshoe kidney, kidney hypo-/dysplasia, duplex collecting system, multicystic kidney dysplasia, and ureter abnormalities [18]. The etiologies of kidney diseases were divided into two types, namely CAKUT and non-CAKUT. Renal anemia was defined as receiving erythropoietin to correct anemia. Mineral bone disease (MBD) was defined as serum calcium (Ca)-phosphate (P) product ≥65 for age ≤12 years, serum Ca-P ≥55 for age >12 years, or elevated parathyroid hormone according to the CKD stage [18]. Hyperuricemia was defined as serum uric acid level ≥5.9 mg/dL for 6- to 8-year-olds, or serum uric acid ≥6.1 mg/dL for 9- to 11-year-olds, or serum uric acid ≥7.0 mg/dL for age ≥12 years in male, or serum uric acid ≥6.2 mg/d for age ≥12 years in female [19]. Hyperlipidemia was defined as serum levels of total cholesterol ≥200 mg/dL, or low-density lipoprotein (LDL) ≥130 mg/dL, or triglyceride (TG) ≥100 mg/dL for 0- to 9-year-olds, and TG ≥130 mg/dL for 10- to 19-year-olds [20].

### 2.3. Anthropometric Measurements

Body height was measured (in cm to the nearest 0.1 cm) with a rigid stadiometer. Body weight was measured (in kg to the nearest 0.1 kg) with an electronic body weight scale. The BMI was calculated as body weight in kilograms divided by the square of body height in meters. BMI percentile for age-and-gender and z-score were determined based on Health Promotion Administration, Ministry of Health and Welfare in Taiwan. Obesity was defined as BMI percentile higher than and equal to the 95th percentile. The fat mass index (FMI), lean body mass index (LBMI), percent body fat (PBF), and the android to gynoid fat ratio (A/G ratio) were measured by DXA (Lunar Prodigy Advance; GE Healthcare, Madison, WI, USA). FMI and LBMI were calculated as both fat mass and lean body mass in kilograms divided by the height in meters squared. FMI for age-and-gender z-scores and LBMI for age-and-gender were based on the reference values underlying the calculator courtesy of the American Journal of Clinical Nutrition [21].

### 2.4. Office BP and ABPM Measurements

Office BP was recorded using a validated and electronic sphygmomanometer after a subject sat for five minutes. High office BP was defined as systolic blood pressure or diastolic blood pressure >95th percentile. The cuff size had a bladder length by a 1:2 to 2:3 width-to-length ratios based on arm circumference. Data from 24-h ABPM were collected for subjects aged 6–18 years using an Oscar II monitoring device (SunTech Medical, Morrisville, NC, USA) as previously reported [4]. The participants and their parents were asked to record activities that may influence BP measurements and awake and sleep periods. The ABPM was programmed to measure at 30 min intervals from 10 pm to 7 am and at 20 min intervals from 7 am to 10 pm. Outlier readings that could be due to artifacts (e.g., systolic BP > 00 mm Hg) were excluded. The criteria of an abnormal ABPM consisted of (1) daytime, nighttime, systolic, or diastolic BPs ≥95th percentile according to gender and height [22]; (2) 25% or greater of SBP or DBP load; and (3) nocturnal BP load dipping <10% compared with average awake BP load. The AASI was calculated by one minus regression slope of diastolic over systolic blood pressure readings from ABPM [23].

### 2.5. Cardiovascular Assessments

Carotid ultrasound and echocardiography were done at the same visit. Carotid ultrasound assessment was performed by an experienced pediatric nephrologist (Pei-Chen Lu) using a ProSound α7 ultrasound device (Aloka Co., Tokyo, Japan). This system consisted of computer assisted analysis software (e-TRACKING system, Aloka Co.) [4]. Pediatric cardiologists performed echocardiographic examination using a Philips IE33 system device (Philips, Bothell, WA, USA). Left ventricular mass index (LVMI) was calculated by Devereux′s formula, as reported previously [24].

### 2.6. Statistical Analysis

All analyses were performed using SPSS version 22.0 (SPSS, Inc, Chicago, IL, USA). Continuous variables with non-normal distribution were presented as medians and interquartile ranges while categorical variables were reported as number (%). The Chi-square test or nonparametric test examined the differences in variables between children with an abnormal ABPM and with a normal ABPM. Multivariate logistic analysis was performed to investigate the associations between risk factors (including age, CKD stage, obesity, hyperuricemia, FMI z-score, and A/G ratio) and an abnormal ABPM. A probability level of *p* < 0.05 indicated statistical significance.

## 3. Results

A total of 101 subjects were enrolled in this study. Table 1 summarizes the clinical, anthropometric, and blood characteristics of all subjects. The median age of study subjects was 10 years and 59.4% of them were boys. The median eGFR at enrollment was 104 mL/1.73 m^2^/min including 70 CKD stage G1 subjects (69.3%), 19 G2 subjects (18.8%), ten G3 subjects (9.9%), and two G4 subjects (2%). CAKUT accounted for 64.4% of the etiologies (*n* = 65). Since 38 subjects were too young to receive the DXA scan and CV assessments, 63 children were grouped from this CKD cohort and defined as the DXA group for subsequent analysis. Participants in this DXA group were older and had higher creatinine levels compared to the whole study group. However, other characteristics were comparable between these two groups.

In the DXA group, 18 subjects (28.6%) were obese. Regarding the complications of CKD, our data showed anemia in one subject (1.6%) with CKD stage G4, mineral bone disease in three subjects (4.8%) with CKD stage G3 or G4, hyperlipidemia in 11 subjects (17.5%), hyperuricemia in 19 subjects (30.2%), and hypertension (by office BP) in 20 subjects (31.7%). These data indicate that almost all participants in this group showed the nature of early or mild-to-moderate CKD.

Unlike office BP, up to 63.5% of subjects (40/63) had an abnormal ABPM. ABPM identified ten patients (15.9%) with 24-h hypertension, 11 patients (17.5%) with daytime hypertension, 17 patients (27%) with nighttime hypertension, 32 patients (50.8%) with increased BP load, and 26 patients (41.3%) with non-dipping nocturnal BP. These subjects were classified by ABPM abnormalities in Table 2. CKD children with abnormal ABPM were older (*p* = 0.006), and showed a larger proportion of CKD stages G2–G4 (*p* = 0.017), obesity (*p* = 0.039), and hyperuricemia (*p* = 0.005).

Then, we determined body composition by DXA. As shown in Table 3, FMI z-score and A/G ratio were higher in children with abnormal ABPM (both *p* = 0.001). However, there was no difference in PBF and LBMI z-score between two groups. Additionally, body composition parameters did not differ statistically between subjects with CKD stage G2–G4 and subjects with G1 (all *p* > 0.05, data not shown), indicating that body composition was not affected by CKD stage in this study. Moreover, we observed that AASI, LVMI, and cIMT in CKD children with abnormal ABPM were comparable with those with normal ABPM.

Using multivariate linear regression analyses, we specified the specific role of risk factors of abnormal ABPM (Table 4). A multivariate linear regression model using the stepwise selection was applied for age, CKD stage, obesity, hyperuricemia, FMI z-score, and A/G ratio. Table 4 shows that only the FMI z-score was independently associated with increased risk of abnormal ABPM (adjusted odds ratio [aOR] 3.00, 95% confidence interval [CI] [1.07–8.46], *p* = 0.037).

## 4. Discussion

In the current study, we provide novel insights into factors involved in subclinical CVD in children with CKD. Our data indicate that body fat plays a key role for an abnormal ABPM in these children. FMI looks like a more trustworthy index than BMI to assess obesity in relation to abnormal ABPM in CKD children as it considers the body composition.

In line with previous reports [3,4,8], our study found that abnormalities on ABPM were highly common in children with CKD. More than 60% of CKD children with early stages had abnormal ABPM profile, while most of them were not found to have hypertension in the out-patient clinic. Our data support the notion that ABPM is superior to office BP readings in detecting hypertension and in predicting cardiovascular events in pediatric CKD [25].

Our study revealed children with an abnormal ABPM were associated with several factors including age, CKD stage, hyperuricemia, obesity, FMI z-score, and A/G ratio. Age-related increases in BP have been observed in adults but not in children [26]. Our regression analysis indicated that age is a confounding factor as two groups being compared have different age distributions. In support of our findings showing that children with CKD stage G2–G4 are prone to exhibit BP abnormalities, existing evidence indicates that the prevalence of hypertension increases as the CKD stage advances in adults as well as in children [2,3,4,27]. Uric acid is not just regarded as a risk factor for CVD, but a valuable therapeutic target also in CKD [28]. Our findings might extend the impact of uric acid on adults to children, even with early stages of CKD. Accordingly, there will be a growing need to better understand whether early targeting on uric acid can prevent the development of CVD in children with CKD, even at an early stage.

Although obesity is a risk factor for the development of CKD, it is paradoxically linked with greater survival in patients with advanced CKD [29]. This obesity paradox may be related to the discrepancy between the uses of BMI and other measures in the definition of obesity. BMI is the most commonly used tool to determine obesity in clinical settings. However, BMI cannot directly measure body fat, by which the discrepancy observed between BMI and other adiposity indices reflects the main limitation of BMI [30]. Accordingly, FMI appears to provide higher accuracy than BMI to assess obesity [30]. Our data demonstrated that the association between high FMI z-score and abnormal ABPM remained significant after adjusting for covariates, while obesity did not. This finding suggests that BMI-defined obesity cannot explain an abnormal ABPM sufficiently, in which body fat is of vital importance. Additionally, our study confirms previous research showing that fat measurements are superior to BMI for predicting CV risk, not just for adults but also for children [31,32,33]. Moreover, examining the relative amount of regional body fat in proportion to other fat regions like A/G ratio is another valid method to test for associations with CV risk [34]. Our study found an association between high A/G ratio and abnormal ABPM in children with CKD, whereas this association did not reach significance after adjusting for other factors. To our knowledge, our results provide the first evidence to show that FMI is associated with CV risk represented by ABPM abnormalities in children with CKD in early stages.

No comparable associations were identified for cardiovascular assessments in our study, despite previous studies reporting that they may increase CV risks in children with CKD [3,6,8]. Given that currently no reference values exist with regard to AASI and cIMT to determine a cut-off value between children with and without CKD, further studies are needed to reveal the relationships using larger populations.

Our study was not without its limitations. First, we acknowledge that the study results may not be generalizable for the entire CKD pediatric population because sampling was performed in one hospital. A multi-center population-based cohort recruiting a large number of patients may be warranted to elucidate the true relationship in the future. Second, considering the long-term nature of childhood CKD, more assessments of body composition with longer follow-ups are required. Third, most participants in our cohort had early stages of CKD. There is a need for further validation of our results in other pediatric cohorts with more advanced CKD. Additionally, dietary intake of salt was not recorded, which comprises a limitation of the present study. Finally, an ethnic difference may be considered because we applied reference values for ABPM, FMI, and LBMI from studies in Europe [21,22].

## 5. Conclusions

Our data provide evidence that the assessment of fat distribution may be useful in determining CV risk in children with CKD in early stages. Considering the exploding obesity epidemic in children, further research is warranted to replicate our findings in other cohorts, monitor body composition parameters with longer-term CV outcomes, and develop effective control at reducing body fat in children with CKD.

## Figures and Tables

**Table 1:** Clinical, biomedical, and anthropometric characteristics of study population.

Characteristics	DXA Group	Total
	*n* = 63	*n* = 101
Age, years	13.4 (10.4–15.7)	10 (5.9–14.9) *
Male	37 (58.7)	60 (59.4%)
CAKUT	36 (57.1%)	65 (64.4%)
CKD stage		
G1	40 (63.5%)	70 (69.3%)
G2	16 (25.4%)	19 (18.8%)
G3	5 (7.9)	10 (9.9%)
G4	2 (3.2)	2 (2%)
Body height, percentile	50 (15–75)	25 (15–50)
Body weight, percentile	64 (35–60)	50 (15–75)
Body mass index, percentile	64 (28–87)	56 (30–80)
Body mass index z-score	0.37 (−0.57–1.11)	0.15 (−0.54–0.84)
Systolic blood pressure, percentile	50 (50–95)	50 (50–93)
Diastolic blood pressure, percentile	50 (50–90)	50 (50–90)
Creatinine, mg/dL	0.6 (0.5–0.86)	0.51 (0.42–0.76) *
eGFR, mL/min/1.73 m^2^	101.4 (80.2–117.1)	104 (83–124)
Urine total protein-to-creatinine ratio, mg/g	64.2 (37.3–410.3)	63.2 (38.4–207.5)
Hemoglobin, g/dL	13.7 (13–14.9)	13.4 (12.7–14.3)
Uric acid, mg/dL	5.7 (4.5–6.7)	5.3 (4.2–6.4)
Sodium, mEq/L	141 (140–142)	141 (140–142)
Potassium, mEq/L	4.3 (4.1–4.5)	4.4 (4.2–4.5)
Calcium, mg/dL	9.5 (9.1–9.9)	9.7 (9.3–9.9)
Phosphate, mg/dL	4.7 (4.3–5.1)	4.9 (4.5–5.3)
LDL-cholesterol, mg/dL	83 (67–102)	87 (70–105)
Total cholesterol, mg/dL	162 (141–185)	163 (146–191)
Triglyceride, mg/dL	67 (53–102)	67 (52–102)

Data given as medians (25th, 75th percentile) or *n* (%). CAKUT = Congenital anomalies of the kidneys and urinary tract. eGFR = Estimated glomerular filtration rate. * *p* < 0.05 by the Chi-square test or the Mann–Whitney *U*-test.

**Table 2:** Comparison of the clinical characteristics and CKD complications in children with CKD stratified for abnormal and normal ABPM profile.

Characteristics	Abnormal ABPM	Normal ABPM	*p* Value
	*n* = 40	*n* = 23	
Age, years	14.4 (11.4–16.4)	11.7 (9.6–14.5)	0.006
Male	27 (67.5%)	10 (43.5%)	0.062
CKD G2–G4	19 (47.5%)	4 (17.4%)	0.017
CAKUT	24 (60%)	12 (52.2%)	0.546
Obesity	15 (37.5%)	3 (13%)	0.039
Mineral bone disease	2 (5%)	1 (4.3%)	0.907
Hyperlipidemia	8 (20%)	3 (13%)	0.484
Proteinuria	13 (32.5%)	6 (26.1%)	0.055
Hyperuricemia	17 (42.5%)	2 (8.7%)	0.005

Data are medians (25th, 75th percentile) or *n* (%). ABPM = 24-h ambulatory blood pressure monitoring. CAKUT = Congenital anomalies of the kidneys and urinary tract.

**Table 3:** Comparison of body composition and CV assessments in children with CKD stratified for abnormal and normal ABPM profile.

	Abnormal ABPM	Normal ABPM	*p* Value
	*n* = 40	*n* = 23	
Body composition			
PBF, %	32.4 (23.7–37.8)	27.5 (23.7–31.7)	0.077
FMI z-score	0.45 (−0.32–1.22)	−0.41 (−0.78–−0.09)	0.001
LBMI z-score	−0.3 (−1.02–0.6)	−0.59 (−1.35–−0.28)	0.084
A/G ratio	0.9 (0.81–1.04)	0.76 (0.72–0.84)	0.001
Cardiovascular assessment			
AASI	0.397 (0.246–0.492)	0.329 (0.243–0.412)	0.084
LVMI, g/m^2^	32.8 (28.6–41.2)	29.8 (26.1–38)	0.253
cIMT, mm	0.4 (0.3–0.4)	0.3 (0.3–0.4)	0.139

Data are medians (25th, 75th percentile) or *n* (%). PBF = Percent body fat. FMI = Fat mass index. LBMI = Lean body mass index. A/G ratio = Android to gynoid fat ratio. AASI = Ambulatory arterial stiffness index. LVMI = Left ventricle mass index. cIMT = Carotid intima-media thickness.

**Table 4:** Adjusted regression model estimates of the association between risk factors and abnormal ABPM.

Factor	*p* Value	aOR	95% CI	
			Lower	Upper
Age	0.313	1.16	0.87	1.53
CKD stage (G2–4 vs. G1)	0.099	3.69	0.78	17.39
Obesity	0.523	0.5	0.06	4.2
Hyperuricemia	0.145	4.24	0.61	29.49
FMI z-score	0.037	3.0	1.07	8.46
A/G ratio	0.296	38.8	0.04	37,000.2

aOR = Adjusted odds ratio. 95% CI = 95% confidence interval. FMI = Fat mass index. A/G ratio = Android to gynoid fat ratio.

## Data Availability

Data will be available upon request.
